# Supplementary material for: Information sharing practices during the COVID-19 pandemic: A case study about face masks
Source: PLoS One. 2022 May 5;17(5):e0268043. doi: 10.1371/journal.pone.0268043 (PMC9071122; doi:10.1371/journal.pone.0268043)
Supplement: S2 Appendix — Tables display the codes used for manual inductive analysis of text and provides definitions. (PDF) [file pone.0268043.s002.pdf]

S2 Appendix: Codebook and Definitions

The following tables display the codes used for manual inductive analysis of text and provide definitions. Tables include: URL Subject Content, Domains, and User Profiles.

URL Subject Content

The URL subject indicates the content of the information to outside sources being shared and/or circulated by Twitter users. Classification made based on the URLs heading and sub-heading. The following themes are those that emerged and were refined by the researchers during the analysis.

Theme	Focus of URL	Sub-theme	Focus of URL
**Art**	Art including paintings and photographs about COVID-19 and/or face mask use.	n/a	n/a
**Cases and face mask use outside the UK**	COVID-19 situation outside the UK including face-mask guidance, outbreaks, political tensions over COVID-19 guidance; and reports on international political/public figures.	Face mask use and guidelines/policies in other countries	Current guidance/policy for face mask use in other countries (not the UK).
		International political/public figure	Reports on international political/public figure in the context of face masks and/or COVID-19 e.g. endorsing or discouraging use of masks.
		Outbreaks and reported cases in other countries	Number of COVID-19 cases, reported cases, or outbreaks in other countries (not the UK).
		Political tensions in/between other countries	Political tensions or disagreements in/between other countries regarding the response to COVID-19.
**COVID-19 (not face mask specific)**	Issues related to COVID-19 but not face-mask specific.	Hoax/overreaction	About someone/people who think COVID-19 is a hoax or the response is an overreaction.
		Mitigation strategies	Other mitigation strategies including social distancing, handwashing and development of vaccine.
		Other	Other issues related to COVID-19 and/or face masks not covered by the sub-themes outlined.
		UK outbreaks and reported cases	Number of COVID-19 cases, reported case, or outbreaks within the UK
**Environmental impact**	Impact of disposable face masks on the environment.	n/a	n/a
**Exemptions and disability concerns**	Who is exempt from face mask use and disability and/or medical concerns, including those with hearing impairments, autism or asthma.	n/a	n/a
**Fact checking**	Claims to check whether statements and information circulated are true.	n/a	n/a
**Guidance and policy**	Information about face mask guidance and or policy, including changes, updates,	Against use and/or guidelines/policy	About someone/people (excluding political/public figures) campaigning against face mask use.

Theme	Focus of URL	Sub-theme	Focus of URL
	how to use, make, a general outline, and calls for clarification about what the guidance is and the enforcement of use. Also includes reports focused on endorsing or against the guidelines.	Call for clarification	Critiques over lack of clarity by the Government about what the guidelines/policy for face mask use are and/or why they have changed.
		Endorsing use and/or stricter guidelines/policy	About someone/people (excluding political/public figures) campaigning for face masks use and/or stricter guidelines/policy.
		Enforcement	Concerns or problems arisen internationally from enforcing the use of face masks.
		Guidance update	Changing guidance (meaning use recommended or not) related to face mask use in the UK.
		How to make	Guidance on how to make a face mask.
		How to use	Guidance on how to wear masks, different types available, washing and skin protection.
		Outline of guidance/policy	Overview of the current guidance/policy for face mask use in the UK.
		Policy update	Changing policy (meaning use mandated) related to face mask use in the UK.
**Invalid link**	URL cannot be accessed	n/a	n/a
**Irrelevant**	URL is irrelevant to COVID-19	n/a	n/a
**Mask use**	Reports on members of the general public using or not using face masks correctly, or reporting issues with their use. For example: receiving abuse, health issues, security concerns and prejudice associated with face coverings.	Abuse for wearing and/or asked to remove	Someone/people receiving abuse for wearing a face mask and/or been asked to remove at work or in public space.
		Health issues from use	Claims of health issues from using face mask
		Mask(s) in use	Masks being used by general members of the public e.g. at an event.
		Mask(s) not being worn or misused	Masks not being worn by general members of the public e.g. at an event or being worn incorrectly.
		Prejudice associated with face coverings or general appearance	Prejudice received before COVID-19 for covering face e.g. for religious purposes and/or prejudice over looks/general appearance either with or without a face mask.
		Security and face recognition	Security concerns regarding use of masks and/or development of face recognition technology.
**PPE supplies**	Lack of availability, scams, manufacturing, quality of personal protection equipment (PPE), particularly for key workers	PPE supplies (International)	Lack of availability, scams, manufacturing, quality of personal protection equipment (PPE), particularly for key workers outside the UK.
		PPE supplies (UK)	Lack of availability, scams, manufacturing, quality of personal protection equipment (PPE), particularly for key workers, within the UK
**Retail**	Websites describing face masks for sale or selling face masks.	Face masks for sale	Description of different face masks for sale or being developed
		Selling face masks	Website selling face masks

Theme	Focus of URL	Sub-theme	Focus of URL
**Scientific evidence and expertise**	Reports on scientific studies and evidence and/or expert opinion related to face-mask use and/or transmission of COVID-19	Expert opinion	Expert opinion about face mask use and/or transmission of COVID-19.
		Scientific studies	Scientific studies and evidence related to face-mask use and/or transmission of COVID-19.
**UK Political and public figures**	Focus on UK public figure(s) e.g. celebrity or political figure(s) in the context of face-masks and/or COVID-19.	Breaking social distancing rules	UK public figure e.g. celebrity or political figure breaking other social distancing rules e.g. unnecessary travel
		Public/political figure against enforcement	UK public figure e.g. celebrity or global political figure saying face mask use should not be or won't be enforced/mandated.
		Public/political figure discouraging use or not wearing	UK public figure e.g. celebrity or political figure discouraging use of or not wearing a face mask.
		Public/political figure encouraging use, stricter guidelines or wearing	UK public figure e.g. celebrity or political figure saying face mask use should not be or won't be enforced/mandated
		Public/political figure infected with COVID-19	UK public figure e.g. celebrity or political figure infected by COVID-19

Domains

Categorising URL domains explores what type of content and information sources are being retweeted or shared. The following table shows the final domain categories that emerged and were refined by the researchers during the analysis.

Domain category	Description of main website content
**Academic/science journal and/or organisation**	Periodical publications with original research articles and/or scientific organisations (including science focused websites) e.g. The Royal Society or Science Magazine and/or academic/scientific institutions e.g. universities
**Broadcasting and radio: international**	International (outside UK) broadcasting channels (tv channels distributing news via audio and video content) which also publish news stories online. e.g CNN. Also includes podcasts.
**Broadcasting and radio: UK**	UK broadcasting channels (tv channels distributing news via audio and video content) which also publish news stories online e.g. BBC. Also includes podcasts.
**Charity, voluntary organisation and/or fundraising.**	Organisation set up to provide help to people in need and/or raising money for charitable initiatives.
**COVID-19 information/statistics**	Data on number of COVID-19 cases and other metrics associated with the pandemic worldwide.
**Fact Checking**	Claims to check whether statements released by the media are true or false.
**Government and public bodies (inc. health agencies)**	Official national governments, local authorities or health agencies e.g. Public Heath England.
**Invalid**	Link broken or invalid.
**Journalism: international print**	Distribution of news stories online by international organisations which also currently print (or previously printed) newspapers and/or magazines in any style.
**Journalism: news aggregator**	Website collating news stories from a range of journalism and broadcasting platforms.
**Journalism: online only**	Distribution of news stories online. Includes independent media outlets with only an online distribution, as well as blogs. Note that websites focusing on science journalism fall under the 'Academic/science journal and/or organisation' category.
**Journalism: UK print**	Distribution of news stories online by UK organisations which also currently print (or previously printed) newspapers and/or magazines in any style.
**Marketing/Branding/Analytics platform**	Offer to help individuals and companies promote themselves and/or analyse number of people visiting website.
**Other**	Content of website does not fall into any of the categories listed.
**Petition/Campaign**	A campaign or petition for change.
**Professional body/Union**	Organisation with individual members practicing a profession and/or association of people with a common interest.
**Retail**	Selling face masks/coverings.
**Search Engine**	Platform for searching internet content.
**Social media**	Enables users to create and share content with one another e.g. Facebook. Note that Twitter was excluded from the analysis.
**Travel**	Information about travel e.g. buses or rail network.
**URL shortening**	Tool for making URL's shorter.

User Profiles

The analysis of user profiles allows for a better understanding of who is circulating information. The following tables shows the final user profile categories that emerged and were refined by the researchers during the analysis.

Individual/organisation	Description
**Individual**	User profile is for an individual person
**Organisation**	User profile is for an organisation or group of people
**n/a**	No indication if individual person or organisation

Profession	Description	
	**Organisation**	**Individual**
**Academia/Research/University**	Online journals, scientific organisations e.g. The Royal Society and/or academic institutions e.g. universities.	Person describing themselves as an academic or researcher. e.g. PhD, Research Associate. Lecturers, Professors.
**Activist/Campaign group**	Group of people of organisation calling for political or social change.	Individual who is part of an activist/campaign group (and identifies this in profile description) and/or explicitly describes themselves as an 'activist' or 'campaigner'.
**Broadcasting/Journalism**	Journalistic or broadcasting platform distributing news stories.	Person working for a journalism or broadcasting organisation and/or describing themselves as a journalist or broadcaster.
**Charity/Volunteer Organisation**	Organisation set up to provide help to people in need.	Someone working/volunteering for charity or volunteer organisation
**Creative Industries**	Organisations representing industries which have an origin in individual creativity e.g. design, music, writing, performing arts (for the purposes of this study excludes radio and tv, which will be categorised as broadcasting/journalism).	Person describing themselves in a profession linked to the creative industries. E.g. artist, musician, author, actor.
**Emergency services**	Organisation responding to emergencies e.g. ambulance service, police, fire brigade.	Emergency responder e.g. firefighter, police or paramedics.
**Government / Government Agency / Local authority**	System governing a country and agencies attached to it. At local level, authority is responsible for public services and facilities in the area.	A member of government, government agencies or local authority e.g.<br>MPs, Mayor, Local Councillor.
**Medical/Healthcare (inc. NHS)**	Organisation with main duties being associated with medicine and/or healthcare e.g. NHS Hospital	Someone working in the medical or healthcare profession e.g. doctor, nurse, surgeon, carer.
**n/a**	No indication of profession in user description.	No indication of profession in user description.

Profession	Description	
	**Organisation**	**Individual**
**Other**	Professional organisation is mentioned in description but does not sit within any of the themes outlined e.g. plumber.	Profession is mentioned in description but does not sit within any of the themes outlined e.g. plumber.
**Professional Association/Union**	Organisation with individual members practicing a profession and/or association of people with a common interest.	Member of a professional association or union.
**Travel**	Specialist in travel e.g. airport, bus company	Person working for a travel organisation.
