# Supplementary material for: Information sharing practices during the COVID-19 pandemic: A case study about face masks
Source: PLoS One. 2022 May 5;17(5):e0268043. doi: 10.1371/journal.pone.0268043 (PMC9071122; doi:10.1371/journal.pone.0268043)
Supplement: Attachment — Submitted filename: Response to reviewers.pdf [file pone.0268043.s005.pdf]

Response to reviewer comments

Manuscript: Information sharing practices during the COVID-19 pandemic: a case study about face masks

We thank the reviewers for the time spent reviewing our manuscript, and we have edited the manuscript to address the points raised. This includes (but is not limited to): ensuring that we set a theoretical context, 2) making our theoretical and practical contributions clearer, 3) shortening the paper to focus on our key findings.

In the following pages we respond to each point raised. Our responses are shown in blue text. We have also provided a copy of the revised manuscript with tracked changes on the submission system.

We feel that these changes have significantly improved the paper, so once again thank you for the time spent to date in the review process. We hope that the manuscript is now ready for publication in PLOS ONE.

Reviewers' comments:

Reviewer's Responses to Questions

Comments to the Author

1. Is the manuscript technically sound, and do the data support the conclusions?

The manuscript must describe a technically sound piece of scientific research with data that supports the conclusions. Experiments must have been conducted rigorously, with appropriate controls, replication, and sample sizes. The conclusions must be drawn appropriately based on the data presented.

Reviewer #1: Yes
Reviewer #2: Yes
Reviewer #3: Yes

---

2. Has the statistical analysis been performed appropriately and rigorously?

Reviewer #1: Yes
Reviewer #2: N/A
Reviewer #3: No

---

3. Have the authors made all data underlying the findings in their manuscript fully available?

The PLOS Data policy requires authors to make all data underlying the findings described in their manuscript fully available without restriction, with rare exception (please refer to the Data Availability Statement in the manuscript PDF file). The data should be provided as part of the manuscript or its supporting information, or deposited to a public repository. For example, in addition to summary statistics, the data points behind means, medians and variance measures should be available. If there are restrictions on publicly sharing data—e.g. participant privacy or use of data from a third party—those must be specified.

Reviewer #1: Yes
Reviewer #2: Yes
Reviewer #3: Yes

---

4. Is the manuscript presented in an intelligible fashion and written in standard English?

PLOS ONE does not copyedit accepted manuscripts, so the language in submitted articles must be

clear, correct, and unambiguous. Any typographical or grammatical errors should be corrected at revision, so please note any specific errors here.
Reviewer #1: Yes
Reviewer #2: Yes
Reviewer #3: Yes

---

5. Review Comments to the Author

Please use the space provided to explain your answers to the questions above. You may also include additional comments for the author, including concerns about dual publication, research ethics, or publication ethics. (Please upload your review as an attachment if it exceeds 20,000 characters)
Reviewer #1: Dear Author(s)

Thank you for the opportunity to read this interesting piece of work. The manuscript is well-written. However, I feel the manuscript is rather long and descriptive falling short of critically engaging with previous research. three major issues need to be addressed. First, the research gap your study attempts to address needs to be expressed more explicitly. Second, the conclusions section failed to engage with previous research. Do your study findings confirm/refute previous research? Finally, the manuscript falls short of discussing the theoretical and practical contributions/implications of study.
Good luck with revising your manuscript.

Thank you for these comments, which by addressing have helped improve the quality of our paper. Our contribution and key findings are now much clearer in the abstract of the paper, as well as the introduction, discussion and conclusions. To highlight the research gap, in the abstract, we now say "*This research examines tweets in this longitudinal context and therefore provides novel insights into the dynamics of crisis communication in an ongoing crisis event with emerging scientific evidence*.". This is re-emphasised in the manuscript.

To focus on the key findings, and therefore the theoretical and practical contributions, we have significantly shortened the paper. Concurrently, the paper has been slightly restructured. We have switched the first and second research questions around, to allow us to discuss the topic modelling and subject categories first and set the scene (i.e. circulation of information includes circulation of scientific evidence and expertise). This also involves restructuring the methodology, results and discussion to keep the correct order. In the discussion section, we have removed text, figures and tables, which we considered to be just descriptive with limited value to our overall arguments (for example, the information on subject sub-categories), as we agree these distracted from the narrative.

To further reduce the length of the paper, we have also merged what were the sections 'changes in the guidance for face masks' and 'knowledge, attitudes, and practices (KAP) towards face masks' into one section: 'Changes in the guidance and use of face masks'. This is now followed by a section entitled 'digital information ecology and crisis communication', which better sets a theoretical context for the work, which we draw upon in the discussion and conclusion sections.

What was the 'Limitations and further work' section has been separated and reduced in length, with the limitations now at the end of the methodology section and further work after the conclusions. This is to further help focus on the key findings and contributions at the end of the paper.

On reflection, we agree that the conclusions did not sufficiently engage with previous work. We have now added references to previous studies and theory in the 'Conclusions and further work' section, in addition to making these clearer in the discussions for each research question. One example of this engagement with previous theory now in our conclusion section is:

"Although existing theoretical frameworks have provided useful approaches for conceptualising user's motivations for accessing information [43,98], their assessments of source credibility [55,58] and organisation risk communication strategies [60], our work highlights that there are specific considerations to be factored in when crisis communication is dependent on emergent scientific evidence. We identified that challenges can arise due to the prolonged circulation out-of-date information, i.e. not strategic misinformation, nor "mis"-information at all, which can have serious ramifications for crisis communication practitioners working to deliver good quality information"

Thank you again for your review.

Reviewer #2: The manuscript presents a very interesting topic, of global scope and likely to interest the world of academia, including sociologists, medical staff, marketers and social media experts, but also people not associated with the scientific community (eg., influencers). It has a universal character, which, however, is not a disadvantage, but deserves recognition. It is not written at a high level of generality, on the contrary, the article is very carefully prepared. The manuscript is presented in an intelligible fashion: the language is clear, correct, and unambiguous. No typographical or grammatical errors were found. The research was well thought out and well designed. The manuscript describes a technically sound piece of scientific research with data that supports the conclusions. The authors have fully provided all data on which the conclusions in the manuscript are based. These data have been provided both within the manuscript and its supplementary information.

We thank you for this really positive feedback. We have acknowledged this in the paper: "*the universal character of our study contributes to developing understanding of the digital information ecology and provides practical insights for crisis communicators."*

Reviewer #3: Review of
Information sharing practices during the COVID-19 pandemic: a case study about face masks
Submitted to PLOS ONE
PONE-D-21- 31236

The paper investigates the sharing of information about face masks during the COVID-19 pandamic in the UK.
In a general sense, there is much to like about the paper. The paper is well written, and the overall argumentation is well crafted.
Nevertheless, I have three substantial issues with respect to the paper that limit the scientific contribution substantially.

Thank you for your detailed comments and the time spend reviewing the manuscript. Please find our responses to each point raised below.

MAJOR ISSUES

(1) CONTRIBUTION:

a. I think that you have an interesting story. However, I was not really convinced about the contribution of the paper. I believe your findings are important, but I really suggest coming up already in the introduction with the main findings. It takes much too long to see the results.

We have added two new paragraphs to the introduction. The first follows the research questions to outline their purpose. The last paragraph briefly describes the main findings:

"Analysing tweets in the longitudinal context of an ongoing crisis such as COVID-19 provides novel insight and understanding into the dynamics and influences of risk crisis communication [8]. The

research, therefore, contributes to developing understanding of the digital information ecology, in particular the circulation of scientific information and expertise in an ongoing crisis context which is subject to uncertainty. This includes insight into when information is shared, including concerns about the recirculation of out-of-date articles, the plurality of information resources and communicators online, including the dominant role of traditional media but also some individuals in information circulation"

We have also made our contribution clearer in the abstract and conclusions section.

b. When reading the abstract, I was wondering about findings and what I can learn from this. You need to be much more specific to "sell" your results.

On reflection, we agree that we could better 'sell' our results. We have therefore re-written the abstract. This includes making our contribution clear in the first sentence and then clearly outlining the key findings in the second paragraph.

c. I also suggest focusing on findings that are (a) new and (b) impactful. For example., I think that it was interesting to discuss the relevance of outdated scientific results (page 32 in the manuscript). This is a key finding! I have no problem if you decide shortening the paper and focus on less but interesting findings.

Thank you for this observation. We have both restructured and shortened the paper to help focus on the key findings i.e. those we consider to be new and impactful. We have switched the first and second research questions around, to allow us to discuss the topic modelling and subject categories first (but in less detail) and set the scene (i.e. circulation of information includes circulation of scientific evidence and expertise). This also involves restructuring the methodology, results and discussion to keep the correct order. In the discussion section, we have removed text which we considered to be just descriptive with limited value to our overall arguments (for example. the information on subject sub-categories), as we agree these distracted from the narrative.

In the discussion section we are clearer about what our new findings are, and the conclusion now highlights three of these: circulation of out-of-date information, points in time that people are seeking information correspond with guidance/policy changes, and the continued dominance of the traditional media despite plurality of voices, although there are examples of high profile scientific organisations and individuals.

d. Many findings that you report are not so surprising and lack (causal) support. You report sharing behavior, but you do not control for many drivers that might lead to specific sharing behavior (e.g., discussion of a news issue on TV, sharing of content by a celebrity etc.). Thus, I wonder if you should reduce the storyline and focus on the really new insights.

In addition to our response to point c, we have also shortened the paper to focus on the key findings by 1) merging what were the sections 'changes in the guidance for face masks' and 'knowledge, attitudes, and practices (KAP) towards face masks' into one section: 'Changes in the guidance and use of face masks'. 2) What was the 'Limitations and further work' section has been separated and reduced in length, with the limitations now at the end of the methodology section and further work after the conclusions. This is to further help focus on the key findings and contributions at the end of the paper. 3) Removed some of the figures and tables which were not necessary to support the key/new insights. 4) Deleted several paragraphs of text which were too descriptive and distracted from the overall narrative.

In our 'further work section' we have acknowledged that further work is required to understand the drivers and motivations behind specific sharing behaviour.

e. Please add a new table that shows in a top-down fashion an overview of all your studies (data, method, and the subsequent findings).

We have added this suggested table to the beginning of the discussion section. Table caption 'Overview of research questions, applicable datasets, content analysed & methods and key findings'. This helps to highlight the key findings and provide a good overview of the study – so thank you for the suggestion.

(2) THEORY: The paper is basically free of theory. While I do not believe that you must add theory to the paper, I suggest presenting insights that are either supported by theory or that substantially challenge theories. Currently, I think your findings are in line with agenda setting theories. Thus, what can we learn? If we cannot learn much for theory testing and building, then I suggest focusing on the managerial impact of your results.

We have restructured the literature review section and now have a section called 'digital information ecology and crisis communication'. We use this section to better provide the theoretical context (with new references), and acknowledge that here are several microenvironments and relevant theories within this digital information landscape:

"*Following Perreault & Perreault's work on the Communication Ecology of Pandemic reporting [38] we acknowledge that there are several microenvironments within the crisis communication ecology, and this study examines how journalistic outputs, citizen commentary, governmental and public health messaging, as the associated communication practices coincide. Each warrants consideration, as the motivations and agendas of different actors in the network contribute to the wider communication ecology. Despite increased efforts to understand the digitally mediated communication practices in both scientific and crisis communication contexts, there is no systematised theorisation that attends to these overlapping and related concerns. Developing this understanding is therefore key for effective crisis communication [39].*"

Within this framing we draw upon relevant theoretical perspectives including Agenda setting, as well as Media Dependence studies and the Social-Mediated Crisis Communication model. We now present insights that either support or challenge some of these theories in the discussion and conclusion. Examples include:

1) "*While studies have developed theoretical standpoints on how to mitigate the effects of misinformation in public health crises [90], what our findings show is that specific challenges can arise due to the prolonged circulation out-of-date information, i.e. not strategic misinformation, nor "mis"-information at all.*"

2) "*As our study found that the distribution of retweets attached to organisations compared to individuals was 58.16% and 41.84% respectively, we agree with previous critiques that is it vital for Social-Mediated Crisis Communication theory to focus on how multiple actors engage with crisis response* [60,62]"

3) "*The shift and increasing focus on guidelines/policy in the 'most retweeted tweets' dataset, particularly after they became mandatory in England's and Scotland's shops, suggests that interest in a topic is linked to the latest news and major events – as covered in Agenda Setting theory, a policy agenda/change has therefore directed the public's attention [52].*"

(3) ROBUSTNESS:

a. I would like to see your findings contrasted to google trends.

We have added google trends data to Figure 2. Alongside the tweet frequency data, we discuss these trends that show people searching for information at particular points in time, in both the results and discussion.

b. Please also show your trend over time and clearly discuss the events that occurred during that time. This allows the reader to better understand confounding events that might drive search (via Google) or tweeting.

We have combined the Google Trends data with the previous diagram we had showing Tweet frequency which includes annotations of key policy changes (Figure 2). We have added arrows to these figures to show the timeframes used in our analysis i.e. TF1. TF2 and TF3. We have also added these arrows, and additional annotations, to the diagram of re-circulated articles (Figure 3) to help the reader better understanding the timeline of events.

c. Please report the reach of the newspapers when discussing the popular domains.

In the section about 'Popular domains' we have added information (and reference to a survey) about the reach of the newspapers. Paragraph beginning 'Within the highest-ranking domains…." After Table 8:

"*Within the highest ranked domains, those categorised as journalism or broadcasting include: The Independent, The Guardian, The Daily Mirror and the BBC. In a survey (conducted April 2019 – March 2020) assessing the monthly reach of leading national newspapers in the United Kingdom [87], The Independent, Guardian and The Daily Mirror are ranked within the top 5 (taking into account phones, tablets, desktops and prints). However, the two highest ranking newspapers in the survey, The Daily Mail and The Sun, only account for 1.17% and 0.96% of total retweets in our 'most retweeted tweets dataset' (ranks 20th and 24th). Therefore, these results suggest that the reach of these newspapers may vary depending on the type of platform being used or topics being discussed*"

d. I think it would also be interesting to discuss why some other relevant posts have not been shared as much (discussion of non-findings).

We have pin-pointed some examples of some information not being shared as much as other posts. For example in the results we state:

"*One external official resource that is noticeable missing from our sample and these conversations about scientific evidence and expertise is SAGE's official website which contains (since 29 May 2020 and with a delay [98]) summaries of their meetings and studies feeding into their discussions [99]. Although in some cases, scientific resources such as The Lancet were drawn upon, official government channels of information about the scientific advice was not to the same extent - it is highly likely this is due to accessibility (i.e. readability) of those documents compared to the overviews/interpretations provided by the media.*".

When talking about the reach of newspapers, we also acknowledge that "*the reach of these newspapers may vary depending on the type of platform being used*", as the Daily Mail and the Sun have the highest reach but do not rank as highly in our analysis.

In our further work section, we have recognised that understanding why posts are shared (or not) is a limitation of our work and state:

"Lastly, we (as have previous studies [47]) discussed that Twitter users are likely to have different motivations for posting and resharing information – however we have not explored these motivations. To truly understand the digital information ecology, including why some posts are shared more than others, and there are differences between people's original tweets and retweeting behaviours, further work on these agendas is welcomed"

Overall, I am supportive towards this paper – but the authors need to streamline the paper to better show their contribution.

I thank you for the opportunity to read this manuscript and I hope that the authors will find the comments helpful and that they will continue to follow this research stream.

---

6. PLOS authors have the option to publish the peer review history of their article (what does this mean?). If published, this will include your full peer review and any attached files.

If you choose "no", your identity will remain anonymous but your review may still be made public.

Do you want your identity to be public for this peer review? For information about this choice, including consent withdrawal, please see our Privacy Policy.
Reviewer #1: No
Reviewer #2: No
Reviewer #3: No

[NOTE: If reviewer comments were submitted as an attachment file, they will be attached to this email and accessible via the submission site. Please log into your account, locate the manuscript record, and check for the action link "View Attachments". If this link does not appear, there are no attachment files.]

While revising your submission, please upload your figure files to the Preflight Analysis and Conversion Engine (PACE) digital diagnostic tool, https://pacev2.apexcovantage.com/. PACE helps ensure that figures meet PLOS requirements. To use PACE, you must first register as a user. Registration is free. Then, login and navigate to the UPLOAD tab, where you will find detailed instructions on how to use the tool. If you encounter any issues or have any questions when using PACE, please email PLOS at figures@plos.org. Please note that Supporting Information files do not need this step.

All figures have been uploaded to PACE and PACE generated files downloaded and used for submission.
